# Non-obese visceral adiposity is associated with the risk of atherosclerosis in Japanese patients with rheumatoid arthritis: a cross-sectional study

**DOI:** 10.1007/s00296-018-4095-0

**Published:** 2018-07-04

**Authors:** Tamami Yoshida, Motomu Hashimoto, Rie Kawahara, Hiroko Yamamoto, Masao Tanaka, Hiromu Ito, Izuru Masuda, Kiminori Hosoda, Wataru Yamamoto, Ryuji Uozumi, Satoshi Morita, Yasutomo Fujii, Tsuneyo Mimori, Kazuko Nin

**Affiliations:** 1 Department of Human Health Sciences, Graduate School of Medicine, Kyoto University, Kyoto, Japan; 2 Department of Advanced Medicine for Rheumatic Diseases, Graduate School of Medicine, Kyoto University, Kyoto, Japan; 3 Department of Orthopedic Surgery, Graduate School of Medicine, Kyoto University, Kyoto, Japan; 4 Medical Examination Center, Takeda Hospital, Kyoto, Japan; 5 Department of Lifestyle-Related Disease, National Cerebral and Cardiovascular Center, Osaka, Japan; 6 Department of Biomedical Statistics and Bioinformatics, Graduate School of Medicine Kyoto University, Kyoto, Japan; 7 Department of Rheumatology and Clinical Immunology, Graduate School of Medicine, Kyoto University, Kyoto, Japan

**Keywords:** Rheumatoid arthritis, Atherosclerosis, Intra-abdominal fat, Adiposity, Cachexia

## Abstract

Rheumatoid arthritis (RA) patients often have altered body composition including reduced muscle mass and increased fat mass. Some RA patients are likely to increase visceral fat without obesity [Body Mass Index (BMI) ≥ 25]. The objective of the study was to determine the association between obesity and/or visceral adiposity and the risk for atherosclerosis in Japanese RA patients. Obesity was evaluated using the BMI, with visceral adiposity evaluated using the visceral fat area (VFA) and the visceral/subcutaneous fat ratio (V/S ratio), quantified using the dual bioelectrical impedance method. Atherosclerosis was evaluated based on the intima–media thickness (IMT) and Plaque score (PS) of the carotid artery, measured using ultrasonography. Multivariate analysis was performed to determine the factors associated with IMT and PS. IMT and PS were compared among groups of patients sub-classified according to BMI and VFA levels. The V/S ratio was higher in RA patients than healthy controls, after adjustment for age, BMI, and waist circumference. On multivariate analysis, the V/S ratio, but not the BMI, was independently associated with the IMT and PS. Among the sub-classifications for BMI and VFA, non-obese patients with a high visceral adiposity (18.5 ≤ BMI < 25 kg/m^2^ and VFA ≥ 100 cm^2^) had the highest IMT (mean IMT, 0.93 ± 0.29 mm; maximum IMT, 1.44 ± 0.71 mm) and PS (1.43 ± 0.61), compared to all other BMI and VFA subgroups. RA patients have increased visceral adiposity, which is associated with a high prevalence of atherosclerotic of plaques. Non-obese RA patients who have visceral adiposity have a specifically higher risk for atherosclerosis.

## Introduction

Patients with rheumatoid arthritis (RA) have a decreased life expectancy compared to the general population due to RA-related health complications and long-term comorbidities [1, 2]. Among these complications, cardiovascular diseases (CVD) are specifically important due to their prevalence among patients with RA and their association with a high mortality rate [3–5]. Atherosclerosis plays an importance role in the development of CVD, with both traditional risk factors (smoking, dyslipidemia, hypertension, physical inactivity, and obesity) and RA-related factors (disease activity and duration) synergistically contributing to the development of atherosclerosis in patients with RA [6].

Obesity is a well-known risk factor of atherosclerosis, with the Body Mass Index (BMI) being a well-established risk factor for CVD [7]. However, CVD in patients with RA is not necessarily associated with obesity, with a low BMI having been associated with CVD events and increased mortality among these patients [8, 9]. Of note is the tendency of patients with a low BMI to have higher disease activity and a worse joint prognosis than patients with a higher BMI [9]. This discrepancy is known as the “obesity paradox”.

One of the reasons for this “obesity paradox” may be a change in body composition due to chronic inflammation in patients with RA. Patients with RA also often develop “rheumatoid cachexia”, defined as a reduction in muscle mass and a concomitant increase in fat mass [10–12]. However, it is not clear whether subcutaneous or visceral fat mass is increased in rheumatoid cachexia [13]. This is an important distinction as an excess accumulation of visceral fat is associated with metabolic abnormalities, such as hypertension, diabetes, and dyslipidemia, which accelerate atherosclerosis and CVD [14, 15]. It is possible that patients with RA who are initially obese may lose weight due to chronic inflammation, achieving a normal BMI despite an increase in visceral fat mass, with an overall increased risk for atherosclerosis. Based on this reasoning, we hypothesized that measurement of the visceral fat mass might better differentiate patients with RA who are at risk for atherosclerosis than the BMI.

To test this hypothesis, we evaluated the association between body composition [BMI, the visceral fat area (VFA) and the visceral-to-subcutaneous fat ratio (V/S ratio)] and atherosclerosis in patients with RA, where the VFA and V/S ratios were quantified using dual biometrical impedance and atherosclerosis using the intima–media thickness (IMT) and Plaque score (PS) of the common carotid artery (CCA), measured with ultrasonography [16, 17]. Traditional atherosclerosis-related factors and RA-related factors were also evaluated and their association to obesity and visceral adiposity tested.

## Methods

This study was a cross-sectional observational study. It adhered to the principles of the Declaration of Helsinki and was approved by the ethics committee of Kyoto University Graduate School and Faculty of Medicine. Approval number: R-357 for RA patients (24 March 2016) and R-0588 for the healthy control group (13 September 2016); informed consent was obtained from all participants.

### Patient study group

We recruited 441 consecutive outpatients with RA from the Kyoto University Rheumatoid Arthritis Management Alliance (KURAMA), between May 1 and November 30, 2016. The inclusion criteria were as follows: age > 18 years; availability of a complete data set; and fulfilment of the American College of Rheumatology/European League against Rheumatism classification 2010 criteria for RA [18]. The following exclusion criteria were applied: presence of an internal or external electronic device; severely degraded health status; presence of fractures and pain preventing assessment of visceral and subcutaneous fat; waist circumference (WC) < 57 cm; poor physical health on the day of examination, defined by the patient’s subjective symptoms; and concurrent cancer and hepatitis treatment, dialysis, and/or sex-hormone suppression or replacement therapy.

### Healthy control group

As a control group, we used the existing data of 1527 of 2085 healthy subjects who underwent a comprehensive medical checkup at Takeda Hospital Medical Examination Center and had complete VFA data available. To be included, patients had to be > 18 years and have a complete data set. The exclusion criteria were as follows: prior or current history of cancer, hepatitis, or dialysis; disease affecting dietary intake; inflammatory, wasting, and/or autoimmune disease; use of steroids and/or sex-hormone suppression or replacement therapy. The patients had provided consent for the use of their anonymized data for research.

### Assessment of atherosclerosis

The IMT and PS for the CCA were evaluated by high-resolution B-mode ultrasonography (Aplio300,500, Toshiba Inc., Japan) using a 12-MHz transducer. The CCA-IMT was measured bilaterally, with patients in the sitting position, 1 cm proximal to the carotid bulb dilatation, with measurements obtained for the far wall of the CCA using an IMT measurement software (Intimascope, Media Cross Co Ltd., Japan) [19]. Plaques were defined as localized elevated lesions with a maximum thickness of more than 1 mm, having a point of inflection on the surface of the intima–media complex. In cases of vascular remodeling, the term plaques was used, irrespective of the presence/absence [20]. For analysis, we used the average IMT of the measurements obtained for the right and left CCA, with plaques included when measuring IMT. The PS was calculated as the total plaque thickness for the available visualization sites in the IMT measurement on the right and left sides.

### Assessment of body composition

Height, weight, WC, and the skeletal muscle ratio (SMR) were measured using standardized protocols. The BMI was calculated as the weight divided the height squared (kg/m^2^). The SMR was measured using a Z impedance analyzer (HBF-701 KARADASCAN, Omron Healthcare Co., Japan) with hand grip and foot plate electrodes [21]. The VFA and subcutaneous fat area (SFA) were measured using a dual bioelectrical impedance analyzer (HDS-2000 DUALSCAN, Omron Healthcare Co., Japan). The previous studies have demonstrated a high correlation coefficient (*r* = 0.888, *p* < 0.001) between VFA measured by a dual bioelectrical impedance analyzer and that measured by abdominal computed tomography (CT) [22].

### Body composition phenotype

According to the definition of obesity and visceral fat in Japan [23], we used a cut-off BMI of 25 kg/m^2^ for obesity and a VFA of 100 cm^2^ for visceral fat. RA patients were classified into five groups as follows: Thinness (+), BMI < 18.5 and VFA < 100; Obesity (−) Visceral adiposity (−), 18.5 ≤ BMI < 25 and VFA < 100; Obesity (−) Visceral adiposity (+), 18.5 ≤ BMI < 25 and VFA ≥ 100; Obesity (+) Visceral adiposity (−), BMI ≥ 25 and VFA < 100; and Obesity (+) Visceral adiposity (+), BMI ≥ 25 and VFA ≥ 100.

### Traditional risk factors for atherosclerosis

The following sociodemographic and lifestyle variables were included in the analysis: age, sex, smoking (current smoker or non-smoker), alcohol intake per week, statin use, family history of premature myocardial infraction (< 65 years), and physical activity level, evaluated using the short version of the International Physical Activity [24]. Hypertension was defined as systolic blood pressure (SBP) ≥ 140 mmHg or diastolic blood pressure (DBP) ≥ 90 mmHg, measured with a blood pressure monitor after a few minutes of resting in a sitting position, or the use of antihypertensive medication. Diabetes was defined as an HbA1c > 6.5% or use of antidiabetic medication. Dyslipidemia was defined as a low-density lipoprotein (LDL-C) ≥ 140 mg/dL or use of statins. The following laboratory variables were included in the analysis: total cholesterol (TC), triglycerides (TG), LDL-C, high-density lipoprotein (HDL-C), and estimated glomerular filtration rate (eGFR).

### RA-related factors

RA disease activity was evaluated using the 28-Joint RA Disease Activity Score (DAS28-ESR) and RA-related physical disability was evaluated using the health assessment questionnaire-disability index (HAQ). Disease status was evaluated using the following laboratory variables: rheumatoid factor (RF), anti-cyclic citrullinated peptide (anti-CCP antibodies), matrix metalloproteinase-3 (MMP-3), C-reactive protein (CRP), and erythrocyte sedimentation rate (ESR). RA treatment included methotrexate (MTX, dosage per week) and the use of biological disease modifying anti-rheumatoid disease drugs (bDMARD) and prednisolone (dosage per day).

### Statistical analysis

Variables with a normal distribution were expressed by their mean ± standard deviation, with variables with a non-normal distribution summarized by their median and range. Comparisons between the RA and control group were performed using *t* tests for continuous variables and Chi-squared test for categorical variables. Propensity score matching was performed to ensure rigorous adjustment for the comparison of fat-related variables. A 1:1 matching ratio was created using the neighbor pair-matching algorithm, with a 0.2 caliper width, adjusting for age, BMI, and WC, by sex. Visceral fat-related variables were compared between the RA and control groups after matching by sex.

The relationship between VFA or BMI and hypertension, diabetes, and dyslipidemia was evaluated for the five levels of VFA and three levels of BMI. Odds ratios (ORs), with the corresponding 95% confidence intervals (CIs), were calculated using logistic regression analysis, with adjustment for the covariates of age, sex, current smoking (+), average daily physical active level, alcohol intake per week, use of bDMARDs, prednisolone and MTX, and the DAS28-ESR score.

An analysis of covariance (ANCOVA) was used to evaluate the effect of each predictor on the mean CCA-IMT and PS. Forward stepwise multivariate analyses were performed to identify the effect of predictors in the presence of other factors associated with the mean CCA-IMT and PS. RA-related variables and metabolic risk factors with a *p* value < 0.05 in the univariate analyses were included in the multivariate analyses. The relationship between atherosclerotic indicators and body composition (BC) phenotype was evaluated using a one-way analysis of variance for continuous variables and Fisher’s exact test for categorical variables, with a Steel–Dwass post-hoc test for multiple comparisons.

For all analyses, a *p* value < 0.05 was considered significant. All statistical analyses were performed using JMP (version 11, SAS Institute Inc., Cary, NC, USA).

## Results

### Characteristics of RA patients

**Fig. 1:**
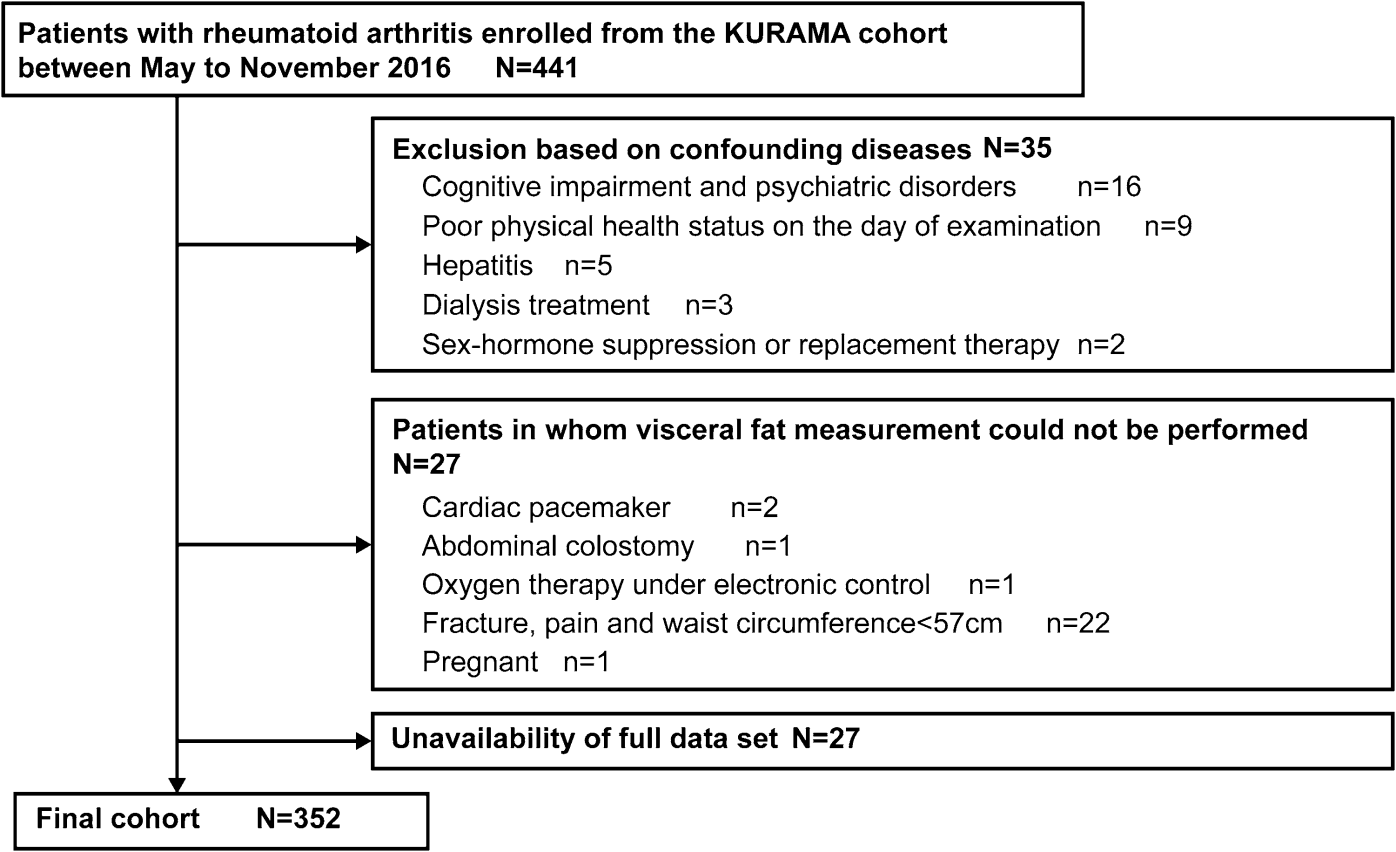
Flow chart of the selection of patients with rheumatoid arthritis in our study group

All 441 consecutive patients enrolled into the study underwent BC measurement and CCA ultrasonography on the same day, together with the evaluation of traditional risk factors for atherosclerosis (diabetes, hypertension, and dyslipidemia, etc.) and RA-related factors (disease activity, etc.). After screening on the exclusion criteria (Fig. 1), the data of 352 patients (292 women, 60 men) were retained for analysis, with an average age of 61.8 years and average RA disease duration of 7 years (Table 1). As MTX, bDMARDs, and prednisolone were used by 72.4, 52.0, and 21.0% of patients, respectively, the mean RA disease activity (DAS28-ESR) was generally low (2.52), with 75.0% of patients being either remission or having low disease activity.

**Table 1:** Demographic and clinical features of patients with RA

	RA patients *N* = 352
Age, years	61.8 (± 11.9)
Male, *n* (%)	60 (17.0)
CCA-meanIMT, mm	0.70 (± 0.16)
CCA-maxIMT, mm	0.91 (± 0.33)
maxIMT ≥ 1.0 mm, *n* (%)	83 (23.6)
Systolic BP, mmHg	123.0 (± 17.1)
Current smoking, *n* (%)	29 (8.2)
Family history of premature CVD, *n* (%)	57 (16.2)
Statin use, *n* (%)	34 (9.7)
Body composition indicators	
BMI, kg/m^2^	22.6 (± 3.6)
VFA, cm^2^	61.9 (± 32.5)
SFA, cm^2^	153.4 (± 70.0)
V/S ratio	0.43 (± 0.19)
Systemic skeletal muscle ratio, %	24.6 (± 3.2)
Waist circumference, cm	84.1 (± 10.1)
Laboratory markers	
Total cholesterol, mg/dL	200.0 (± 33.3)
HDL cholesterol, mg/dL	70.7 (± 18.7)
LDL cholesterol, mg/dL	115.0 (± 28.4)
Triglycerides, mg/dL	88.7 (± 45.0)
eGFR, mL/min/1.73 m^2^	18.1 (± 18.1)
Comorbidities	
Hypertension, *n* (%)	113 (32.1)
Diabetes, *n* (%)	29 (8.2)
Dyslipidemia, *n* (%)	130 (36.9)
RA disease characteristics	
RA duration, years	7 (0.7–46)
RF, IU/mL	39.0 (8-2833.6)
Anti-CCP, U/mL	51.5 (0.6–3260)
CRP, mg/dL	0.1 (0.1–9.6)
DAS28-ESR	2.52 (0.33–7.20)
High, *n* (%)	2 (0.6)
Moderate	86 (24.4)
Low	76 (21.6)
Remission	188 (53.4)
Stage 1/2, *n* (%)	103 (29.3)/95 (27.0)
3/4	64 (18.1)/90 (25.6)
Class I/ II, *n* (%)	188 (53.4)/146 (41.5)
III/ IV	18 (5.1)/0
Current RA therapeutics	
MTX use, *n* (%)	255 (72.4)
MTX dosage, mg/week	8 (2–16)
Other cs DMARD use, *n* (%)	120 (34.0)
ts DMARD use, *n* (%)	2 (0.01)
Biological agent use, *n* (%)	183 (52.0)
Prednisolone use, *n* (%)	74 (21.0)
Prednisolone dosage, mg/day	4 (1–10)

Values are shown as mean (± SD) or number (percentage). Values of RA disease characteristics and current RA therapeutics are shown as median (range)

*RA* rheumatoid arthritis, *BMI* Body Mass Index, *VFA* visceral fat area, *SFA* subcutaneous fat area, *V/S ratio* visceral fat area/subcutaneous area ratio, *BP* blood pressure, *CVD* cardiovascular disease, *HDL* high-density lipoprotein, *LDL* low-density lipoprotein, *eGFR* estimated glomerular filtration rat, *RF* rheumatoid factor, *anti-CCP* anti-cyclic citrullinated peptide, *CRP* C-reactive protein, *DAS28-ESR* 28-joint Disease Activity Score using erythrocyte sedimentation rate, *MTX* methotrexate, *cs DMARD* conventional synthetic disease modifying anti-rheumatic drugs, *ts DMARD* targeted synthetic DMARD

### Comparison of body composition between the RA and control group

BC was compared between the RA and the control group, with adjustment for age, BMI, and WC (Table 2). After matching, 285 individuals from each group were included in the analysis (226 women and 59 men). Overall, there was no between-group difference in VFA. However, the proportion of females in the highest VFA classification (VFA ≥ 100 cm^2^) was higher in the RA than control group (8.4 versus 3.1%, respectively; *p* = 0.015). The V/S ratio was higher in the RA than control group, even after adjustment for BMI and WC, for both females (0.39 ± 0.18 versus 0.34 ± 0.13; *p* = 0.002) and males (0.58 ± 0.16 versus 0.51 ± 0.13; *p* = 0.013). This result suggests that the visceral fat mass distribution was higher for the RA than control group.

**Table 2:** Comparison of visceral fat-related variables between patients with RA and the control group, before and after propensity score matching

Sex	Variables	Before matching			After matching*		
		RA patients	Controls	*p* value^†^	RA patients	Controls	*p* value^†^
Women	*n*	292	482		226	226	
	Age, years	61.4 ± 12.2	53.9 ± 11.7	< 0.001	58.7 ± 11.8	58.7 ± 11.8	0.959
	Body Mass Index, kg/m^2^	22.3 ± 3.7	23.0 ± 3.4	0.012	22.6 ± 3.8	22.6 ± 3.0	0.925
	WC, cm	83.3 ± 10.2	82.0 ± 9.1	0.074	82.9 ± 0.6	82.9 ± 0.6	0.976
	VFA, cm^2^	56.0 ± 28.1	53.1 ± 27.0	0.145	55.3 ± 29.0	53.8 ± 23.7	0.538
	V/S ratio	0.40 ± 0.18	0.33 ± 0.15	< 0.001	0.39 ± 0.18	0.34 ± 0.13	0.002
	VFA ≥ 100 cm^2^, *n* (%)	25 (8.6)	23 (4.8)	0.034	19 (8.4)	7 (3.1)	0.015
	SFA, cm^2^	152.5 ± 72.9	173.1 ± 70.4	< 0.001	156.3 ± 75.2	168.6 ± 65.9	0.064
Man	*n*	60	1045		59	59	
	Age, years	63.8 ± 10.6	52.7 ± 10.9	< 0.001	64.5 ± 9.1	64.3 ± 9.4	0.905
	Body Mass Index	24.2 ± 3.2	24.2 ± 2.9	0.998	24.3 ± 3.0	24.7 ± 3.2	0.481
	WC, cm	88.1 ± 1.0	86.3 ± 0.3	0.084	88.6 ± 8.4	89.7 ± 7.6	0.459
	VFA, cm^2^	90.3 ± 37.5	79.0 ± 34.5	0.015	91.7 ± 36.4	89.8 ± 35.0	0.778
	V/S ratio	0.57 ± 0.17	0.48 ± 0.16	< 0.001	0.58 ± 0.16	0.51 ± 0.13	0.013
	VFA ≥ 100 cm^2^, *n* (%)	23 (38.3)	232 (22.2)	0.004	23 (39.0)	22 (37.3)	0.850
	SFA, cm^2^	158.2 ± 53.9	163.1 ± 53.9	0.450	159.7 ± 53.1	174.4 ± 54.5	0.139

Values are shown as mean ± SD or number (percentage)

*WC* waist circumference, *VFA* visceral fat area, *V/S* visceral fat area/subcutaneous area, *SFA* subcutaneous fat area

*The matching ratio is 1:1 with adjusting for age, Body Mass Index and waist circumference

^†^ *t* test for continuous variables and Chi-squared test for categorical variables

### Association between obesity and visceral adiposity with traditional risk factors for atherosclerosis

The association between obesity and visceral adiposity with traditional risk factors of atherosclerosis (hypertension, diabetes, and dyslipidemia) was evaluated for the 352 patients in the RA group across the 3 BMI levels (BMI-1; BMI < 18.5, BMI-2; 18.5 ≤ BMI < 25, and BMI-3; BMI ≥ 25) and 5 visceral adiposity levels (VFA-1; VFA < 25, VFA-2; 25 ≤ VFA < 50, VFA-3; 50 ≤ VFA < 75, VFA-4; 75 ≤ VFA < 100, and VFA-5; VFA ≥ 100). With regard to BMI, the incidence of hypertension and dyslipidemia was higher in the BMI-3 than either BMI-1 or BMI-2 levels (Table 3). Similarly, the incidence of hypertension, diabetes, and hyperlipidemia was higher in the VFA-5 level than any other of the 4 VFA levels (VFA-1 through VFA4; Table 3). The CCA-IMT and PS also increased with the degree of obesity and adiposity (Table 3). This result suggests that both obesity and visceral adiposity are associated with traditional risk factors of atherosclerosis, with patients in the highest BMI (BMI ≥ 25) and VFA (VFA ≥ 100) levels being at highest risk of atherosclerosis.

**Table 3:** Multivariate analyses for metabolic syndrome-related disease by each stage of VFA and BMI

*N* = 352	Hypertension (*n* = 113)				Diabetes (*n* = 29)				Dyslipidemia (*n* = 130)				Mean IMT	PS
	OR	Lower CI	Upper CI	*p* value	OR	Lower CI	Upper CI	*p* value	OR	Lower CI	Upper CI	*p* value	Average (SD)	Average (SD)
BMI stage														
BMI-1 (*n* = 37)	1.00	Reference			1.00	Reference			1.00	Reference			0.67 (0.12)	0.00 (0.00)
BMI-2 (*n* = 236)	0.86	0.36	2.20	0.748	1.94	0.34	36.69	0.505	2.11	0.92	5.36	0.080	0.69 (0.17)	0.20 (0.05)
BMI-3 (*n* = 79)	4.28	1.63	12.00	0.003	5.56	0.92	108.02	0.063	4.97	1.96	13.67	0.001	0.72 (0.14)	0.33 (0.17)
VFA stage														
VFA-1 (*n* = 36)	1.00	Reference			1.00	Reference			1.00	Reference			0.66 (0.12)	0.03 (0.03)
VFA-2 (*n* = 102)	0.79	0.29	2.26	0.646	0.98	0.12	20.67	0.989	1.13	0.48	2.77	0.774	0.65 (0.12)	0.12 (0.06)
VFA-3 (*n* = 117)	1.06	0.41	2.98	0.901	1.60	0.24	31.48	0.661	0.95	0.41	2.30	0.902	0.70 (0.15)	0.14 (0.08)
VFA-4 (*n* = 49)	2.04	0.70	6.27	0.193	4.27	0.66	84.33	0.140	1.70	0.65	4.61	0.282	0.72 (0.20)	0.07 (0.05)
VFA-5 (*n* = 48)	4.07	1.36	13.06	0.012	6.75	1.07	132.51	0.041	3.76	1.38	10.81	0.009	0.77 (0.19)	0.85 (0.31)

Covariates: age, sex, current smoking (+), average daily physical active level, alcohol intake per week, biologics use, prednisolone use, methotrexate use, and DAS28-ESR

VFA-1; VFA < 25, VFA-2; 25 ≤ VFA < 50, VFA-3; 50 ≤ VFA < 75; VFA-4; 75 ≤ VFA < 100, VFA-5; VFA ≥ 100. BMI-1; BMI < 18.5, BMI-2; 18.5 ≤ BMI < 25, BMI-3; BMI ≥ 25

*OR* odds ratio, *CI* 95% confidence interval, *SD* standard deviation, *VFA* visceral fat area (cm^2^), *BMI* Body Mass Index

### Factors associated with atherosclerosis in patients with RA

The association between obesity and visceral adiposity and CCA-IMT and PS is presented in Table 4. On univariate analysis, CCA-IMT was positively associated with traditional risk factors for atherosclerosis, including SBP, TC, LDL-C, diabetes, BMI, and visceral adiposity (VFA, V/S ratio) while negatively associated with eGFR and HDL-C. On multivariate analysis, the V/S ratio, but not BMI, was independently associated with CCA-IMT (Table 4). Similarly, PS was also positively associated with traditional risk factors for atherosclerosis, including current smoking and visceral adiposity (VFA, V/S ratio) while negatively associated with HDL-C. In addition to the traditional risk factors, RA-related factors, including disease activity (DAS-ESR), MMP-3, and the use of glucocorticoids were also positively associated with PS. On multivariate analysis, sex (male), the V/S ratio and the DAS28-ESR score were independently associated with PS. This result suggests that visceral adiposity (V/S ratio) is a stronger indicator of atherosclerosis than obesity (BMI) in patients with RA.

**Table 4:** Univariate and multivariate analyses for independent factors associated with the meant IMT and Plaque score in patients with RA

Dependent variable	Independent variables	Univariate			Multivariate			
		*β*	95% CI	*p*	*β*	95% CI	*p*	Standardized *β*
Mean IMT	Age (10 years)	0.073	0.062 to 0.085	< 0.001	0.063	0.051–0.075	< 0.001	0.474
	Sex (male)	0.081	0.038 to 0.124	< 0.001	0.050	0.012–0.088	0.01	0.119
	Body Mass Index (1 unit)	0.006	0.002 to 0.012	0.009				
	WC (cm)	0.003	0.001 to 0.004	0.001				
	VFA (10 cm^2^)	0.011	0.006 to 0.016	< 0.001				
	V/S ratio (per 0.1)	0.016	0.007 to 0.025	< 0.001	0.008	< 0.001–0.016	0.037	0.096
	Systemic SMR (%)	− 0.008	− 0.013 to − 0.003	0.002				
	Systolic BP (10 mmHg)	0.028	0.018 to 0.037	< 0.001	0.009	< 0.001–0.018	0.032	0.100
	eGFR (mL/min/1.73 m^2^)	− 0.002	− 0.003 to − 0.001	< 0.001				
	TC (10 mg/dL)	0.008	0.003 to 0.013	0.001				
	LDL-C (10 mg/dL)	0.012	0.007 − 0.019	< 0.001	0.007	0.002–0.012	0.004	0.130
	HDL-C (10 mg/dL)	− 0.010	− 0.018 to − < 0.001	0.038				
	Triglycerides (10 mg/dL)	0.006	0.003 to 0.010	< 0.001				
	Diabetes (+)	0.101	0.041 to 0.160	0.001				
Plaque score	Age (10 years)	0.090	< 0.001 to 0.171	0.049				
	Sex (male)	0.543	0.277 to 0.809	< 0.001	0.489	0.208–0.770	< 0.001	0.189
	VFA (10 cm^2^)	0.050	0.018 to 0.081	0.002				
	V/S ratio (per 0.1)	0.089	0.036 to 0.142	0.001	0.057	0.002–0.112	0.043	0.111
	Systemic SMR (%)	0.034	0.003 to 0.066	0.033				
	HDL-C (10 mg/dL)	− 0.066	− 0.121 to − 0.012	0.017				
	Current smoking (+)	0.478	0.110 to 0.847	0.011				
	DAS28-ESR (1 unit)	0.108	0.001 to 0.216	0.049	0.133	0.028–0.238	0.014	0.129
	MMP-3 (10 ng/mL)	0.010	0.002 to 0.023	0.025				
	PSL dosage (mg/dL/day)	0.067	0.014 to 0.120	0.013				

*N* = 352. Units in parentheses are units for *β* as estimated values

Independent variables (in mean IMT and Plaque score): RA-related variables and metabolic risk factors with a *p* value < 0.05 in the univariate analyses are described

Covariate in multivariate analyses: covariates are variables in which numerical values are listed. These covariates were selected using a stepwise method use *p* < 0.05 for (rather than in) independent variables

*CI* confidence interval, *standardized β* standardized partial regression coefficient, *VIF* variance inflation factor, *WC* waist circumference, *VFA* visceral fat area, *V/S* visceral fat area/subcutaneous area, *SMR* skeletal muscle ratio, *BP* blood pressure, *eGFR* estimated glomerular filtration rate, *TC* total cholesterol, *LDL-C* low-density lipoprotein cholesterol, *HDL-C* high-density lipoprotein cholesterol, *DAS28-ESR* 28-joint Disease Activity Score using erythrocyte sedimentation rate, *MMP-3* matrix metalloproteinase-3, *PSL* prednisolone

No significant associations were observed between the atherosclerotic indicators (CCA-IMT and PS) and the SFA, diastolic blood pressure, physical active level, family history of CVD, RA disease duration, RF, anti-CCP, CRP, use of specific bDMARDs (TNF inhibitor), MTX dosage, or statin therapy.

### Relationship between body composition phenotype and the risk of atherosclerosis

The CCA-IMT and PS and risk factors for atherosclerosis were evaluated across the five classifications of BC phenotype (Table 5). Both CCA-mean IMT and PS were remarkably higher in the Obesity (−) Visceral adiposity (+) group than any other groups (mean ± SD, 0.93 ± 0.29 for CCA-mean IMT and 1.43 ± 0.61 for PS). The CCA-IMT and PS were similar among the other groups, including the Obesity (+) Visceral adiposity (+) and Thinness (+) groups. Notably, RA disease activity was higher in the Thinness (+) group (mean ± SD, 3.24 ± 1.03) than any other group (*p* = 0.047). Therefore, non-obese patients with RA who have visceral adiposity are specifically at high risk for atherosclerosis, with patients in the Thinness (+) group having higher disease activity than patients in the normal and obese groups.

**Table 5:** Characteristics of RA patients according to body composition phenotype combined with VFA and BMI

*N* = 352	BMI < 18.5	18.5 ≤ BMI < 25		BMI ≥ 25		*p* value*
	VFA < 100	VFA < 100	VFA ≥ 100	VFA < 100	VFA ≥ 100	
	Thinness (+) *n* = 37 (10.5%)	Obesity (−) Visceral adiposity (−) *n* = 224 (63.6%)	Obesity (−) Visceral adiposity (+) *n* = 12 (3.4%)	Obesity (+) Visceral adiposity (−) *n* = 45 (12.8%)	Obesity (+) Visceral adiposity (+) *n* = 34 (9.7%)	
Age (years)	61.3 ± 13.3	61.1 ± 12.1	71.8 ± 6.7	62.1 ± 11.7	62.3 ± 9.6	0.054
Male, *n* (%)	1 (2.7)	31 (13.8)	7 (58.3)	6 (13.3)	15 (20.6)	0.002
Mean IMT (mm)	0.67 ± 0.12	0.68 ± 0.15	**0.93 ± 0.29** ^†^	0.73 ± 0.16	0.72 ± 0.11	< 0.001
Max IMT (mm)	0.85 ± 0.17	0.88 ± 0.32	1.44 ± 0.71^‡^	0.97 ± 0.30	0.91 ± 0.17	< 0.001
Max IMT 1.0 mm or more, *n* (%)	7 (18.9)	46 (20.5)	10 (83.3)	11 (24.4)	9 (26.5)	< 0.001
Plaque score	0.0 (0)	0.13 ± 0.04	**1.43 ± 0.61** ^§^	0.38 ± 0.26	0.28 ± 0.19	< 0.001
Body composition indicators						
VFA (cm^2^)	33.7 ± 15.0	50.6 ± 19.1	113.8 ± 13.6	78.2 ± 15.8	126.8 ± 26.6	< 0.001
SFA (cm^2^)	69.6 ± 25.7	137.7 ± 48.0	156.9 ± 39.8	228.4 ± 57.3	247.8 ± 66.7	< 0 0.001
V/S ratio	0.50 ± 0.22	0.40 ± 0.17	**0.76 ± 0.16** ^||^	0.36 ± 0.13	**0.54 ± 0.17** ^¶^	< 0.001
Waist circumference (cm)						
Man	63.0 ± 0	83.4 ± 6.0	87.9 ± 3.9	92.3 ± 7.0	98.0 ± 5.7	< 0 0.001
Women	71.2 ± 5.1	81.5 ± 7.3	92.4 ± 4.5	94.0 ± 7.1	100.5 ± 6.8	< 0 0.001
Systemic skeletal muscle ratio (%)	25.5 ± 3.3	24.8 ± 3.2	25.8 ± 3.3	23.1 ± 2.5	24.7 ± 3.6	0.006
Comorbidities						
Hypertension, *n* (%)	9 (24.3)	54 (24.1)	6 (50.0)	22 (48.9)	22 (64.7)	< 0 0.001
Diabetes, *n* (%)	1 (2.7)	10 (4.5)	5 (41.7)	6 (13.3)	7 (20.6)	< 0 0.001
Dyslipidemia, *n* (%)	8 (21.6)	73 (32.6)	7 (58.3)	21 (46.7)	21 (61.8)	< 0 0.001
RA disease characteristics						
Duration (year)	11.1 ± 7.8	10.6 ± 10.0	11.0 ± 8.7	11.9 ± 10.1	8.9 ± 7.3	0.746
HAQ score	0.66 ± 0.62	0.46 ± 0.56	0.69 ± 0.69	0.48 ± 0.59	0.51 ± 0.60	0.256
CRP (mg/dL)	0.66 ± 1.81	0.35 ± 0.83	0.33 ± 0.38	0.26 ± 0.33	0.38 ± 0.56	0.329
DAS28-ESR	3.24 ± 1.03**	2.61 ± 0.91	2.84 ± 0.87	2.77 ± 1.02	2.23 ± 0.89	0.047
Current RA therapeutics characteristics						
cs DMARD use, *n* (%)	32 (86.4)	196 (87.5)	9 (75.0)	37 (82.2)	27 (79.4)	0.416
ts DMARD use, *n* (%)	0 (0)	2 (0.9)	0 (0)	0 (0)	0 (0)	1.000
Biological agent use, *n* (%)	21 (56.8)	115 (51.3)	6 (50.0)	22 (48.9)	19 (55.9)	0.945
Prednisolone use, *n* (%)	9 (24.3)	37 (16.5)	4 (33.3)	14 (31.1)	10 (29.4)	0.061

Data are expressed as mean ± SD, or number (percentage)

*BMI* Body Mass Index, *VFA* visceral fat area, *IMT* intima–media thickness, *SAF* subcutaneous fat, *V/S* visceral fat area/subcutaneous fat area, *HAQ* Health Assessment Questionnaire, *CRp* C-reactive protein, *DAS28-ESR* Disease Activity Score in 28 joints using erythrocyte sedimentation rate, *csDMARD* conventional synthetic disease modifying anti-rheumatic drugs, *ts DMARD* targeted synthetic DMARD

*Analysis of variance for continuous variables or Fisher’s exact test for categorical variables

^†‡§||^ *p* < 0.05 for pairwise comparison with all other groups (multiple comparisons using Steel–Dwass)

^¶^ *p* < 0.05 for pairwise comparison with the group of Obesity (−) Visceral adiposity (−) and Obesity (+) Visceral adiposity (−), *p* = < 0.001, *p* = < 0.001, respectively (multiple comparisons using Steel–Dwass)

**p < 0.05 for pairwise comparison with the group of Obesity (−) Visceral adiposity (−) and Obesity (+) Visceral adiposity (+), *p* = 0.045, *p* = 0.007, respectively (multiple comparisons using Steel–Dwass)

## Discussion

Our evaluation of the association between obesity and visceral adiposity and risk of atherosclerosis in patients with RA revealed a higher VFA in patients with RA than a healthy control group, with the visceral adiposity being an independent predictor of atherosclerosis in these patients. Moreover, we identified a specific risk for atherosclerosis among non-obese patients with RA patients having high visceral adiposity. Although the association between obesity, adiposity, and the risk of atherosclerosis has previously been evaluated [25, 26], our study is the first to have evaluated BC and atherosclerosis simultaneously in a cohort of patients with RA patients, including potential atherosclerosis-related and RA-related confounding factors.

Our finding of an increase in visceral fat mass in the RA group compared to the control group is consistent with the previous reports of a higher VFA or trunk fat in patients with RA than a control group [25, 26]. Although a WC ≥ 85 cm in females and 90 cm in males is generally used to screen for increased visceral fat mass in Japanese patients based on the definition of obesity and visceral fat obesity in Japan [23]. This is different from the cut-off value in Europe (80 cm for females and 94 cm for males) and in the United States (88 cm for females and 102 cm for males). Our findings indicate that WC might not be sufficient as it is the V/S ratio which was specifically increased between the RA and control group, even after adjustment for BMI and WC. Therefore, measurement of the visceral fat mass (VFA or V/S ratio) may be more suitable to differentiate patients at risk for atherosclerosis and CVD events than the BMI. In fact, on multivariate analysis, the V/S ratio but not BMI remained as an independent factor associated with atherosclerosis in the RA group. Of note, patients with a normal BMI but a VFA > 100 cm^2^ [Obesity (−), Visceral adiposity (+) group] had particularly high CCA-IMT and PS compared to the other groups. Adipose tissue produces and secretes biologically active substances, collectively called adipocytokines, which include both pro-atherosclerotic and anti-atherosclerotic substances [27]. The dysregulation of adipocytokines may contribute to the pathogenesis of atherosclerosis in patients with a higher V/S fat ratio [28].

Our result is conceptually consistent that patients with RA have a unique body composition, called “rheumatoid cachexia”. In general, “cachexia” is a complex metabolic syndrome associated with underlining illness and characterized by loss of body weight and loss of muscle mass and fat mass [29]. In contrast, in RA, muscle wasting is a common feature, but the fat mass is preserved, or even increased, such that a low BMI is uncommon in these patients. Therefore, two types of cachexia appear to exist among patients with RA [13], namely, “classical cachexia”, characterized by a low BMI and low VFA (Thinness (+) group in our study), and “rheumatoid cachexia”, characterized by a normal BMI with increased adipose tissue [Obesity (−) Visceral adiposity (+) group in our study]. The latter might also be called for “sarcopenic obesity” [30, 31] or “visceral fat obesity”. In our study, patients with “classical cachexia” [Thinness (+)] show higher disease activity, while the patients with “rheumatoid cachexia” [Obesity (−) Visceral adiposity (+)] show high risk of atherosclerosis. Because patients with RA may lose body weight due to chronic inflammation [32], our Obesity (−) Visceral adiposity (+) group might include patients who were initially obese, and, therefore, at high risk for atherosclerosis, and lost weight due to chronic inflammation. However, these speculations will need to be carefully evaluated through longitudinal studies in the future.

Whereas “classical cachexia” can be easily diagnosed by a low BMI, “rheumatoid cachexia” with normal BMI and increased visceral fat mass is difficult to detect by routine clinical examination, with WC being an insufficient measure. Measurement of the VFA and V/S ratio by bioelectrical impedance method may be useful to identify patients at real risk for atherosclerosis and CVD events. The European League against Rheumatism for CVD risk management among patients with RA patients recommended assessment of both traditional CVD risk factors and RA-specific factors [33] and screening for asymptomatic atherosclerotic plaques by use of carotid ultrasound as part of the CVD risk evaluation [34]. We propose that the evaluation of visceral fat is important to improve the management of atherosclerosis and CVD in patient with RA.

Limitations of our study include the use of bioelectrical impedance, rather than the gold standard abdominal CT, for the measurement of the VFA and V/S ratios, to lower the exposure to ionized radiation and recruitment of patients from a single institution, increasing the risk for selection bias and the inclusion of only Japanese individuals, who are generally thinner and with a lower risk for CVD event compared to Europeans (based on the Organization for Economic Co-operation and Development’s data). Of note, elderly patients with RA, who tend to have more joint symptoms, were not included in our study, which would limit the generalization of our findings. Despite these limitations, we consider that our findings adequately reflect the patients with RA in a typical clinical practice.

In conclusion, the accumulation of visceral fat along with a normal BMI was associated with a high prevalence of atherosclerotic plaques in patients with RA. Therefore, evaluation of the VFA may be useful for assessing CVD risk in these patients. Effective management of the risk for atherosclerosis and CVD events to improve life expectancy of patients with RA is important as advances of RA therapies are improving the control over disease activity and joint outcomes. Precise identification would improve this risk management.
